# CCL5 Release by CCR9+ CD8 T Cells: A Potential Contributor to Immunopathology of Primary Sjögren’s Syndrome

**DOI:** 10.3389/fimmu.2022.887972

**Published:** 2022-06-01

**Authors:** Anneline C. Hinrichs, Sofie L. M. Blokland, Aike A. Kruize, Floris P. J. Lafeber, Helen L. Leavis, Joel A. G. van Roon

**Affiliations:** ^1^ Department of Rheumatology & Clinical Immunology, University Medical Center Utrecht, Utrecht University, Utrecht, Netherlands; ^2^ Center for Translational Immunology, University Medical Center Utrecht, Utrecht University, Utrecht, Netherlands

**Keywords:** primary Sjögren’s syndrome (pSS), CCR9+ T cells, autoimmunity, CCL5, CD8 T cells, transcriptomics

## Abstract

**Introduction:**

Increased CCL5 expression and CD8 T cells have been shown to be pivotal regulators of immunopathology in primary Sjögren’s syndrome (pSS) and pSS-like disease. Increased CCL5 expression by CCR9+ CD4 T cells has previously been implicated as a contributor to immunopathology in pSS. The role of CD8 T cells and in particular CCR9+ CD8 T cells and their potential to secrete CCL5 has not previously been studied in pSS. In this study we investigated both CCR9 and CCL5 expression by CD8 T cells in pSS patients compared to healthy controls (HC).

**Methods:**

CCR9 expression on CD8 T cells from peripheral blood was compared between patients with pSS and HC by flow cytometry. Intracellular CCL5 expression by naive, memory and effector CCR9- and CCR9+ CD8 T cells was assessed. In addition, the capacity and pace of CCL5 release upon T cell activation was determined for all subsets and compared with CD4 T cells.

**Results:**

The frequency of circulating CCR9+ CD8 T cells in pSS patients is increased compared to HC. Antigen-experienced CD8 T cells, especially CCR9+ effector CD8 T cells, express the highest CCL5 levels, and release the highest levels of CCL5 upon activation. Memory and effector CD8 T cells of pSS patients express significantly less CCL5 and subsequently release less CCL5 upon stimulation compared to HC. CCR9+ CD8 T cells rapidly release CCL5 and significantly more than CCR9+ CD4 T cells.

**Conclusion:**

CCR9+ CD8 T cells express more CCL5 than CCR9- CD8 T cells. CCL5 is rapidly released upon activation, resulting in reduced intracellular expression. Reduced CCL5 expression by an elevated number of antigen-experienced CCR9-expressing CD8 T cells in pSS patients points towards increased release *in vivo*. This suggests that CCL5 release by CCR9+ CD8 T cells contributes to immunopathology in pSS.

## Introduction

Primary Sjögren’s syndrome (pSS) is a chronic autoimmune disorder, associated with inflammation of the exocrine glands and in some cases other organs, resulting in complaints of dryness of eyes and mouth and extra-glandular manifestations (1). Biopsies of salivary gland tissue of pSS patients show lymphocytic aggregates, mainly consisting of CD4 T cells, CD8 T cells and B cells (1–4). However, also elevated numbers of cell types such as NK cells and NK T cells have been demonstrated in pSS salivary glands (5–7). B cell hyperactivity is a hallmark feature of the disease, which is reflected by the production of autoantibodies, elevated serum IgG and the increased risk of developing lymphoma (1, 8).

T follicular helper (Tfh) cells are important contributors to B cell hyperactivity. Both Tfh cell numbers and typical Tfh-associated cytokines (e.g. IL-21, IL-4 and CXCL13) are increased in the salivary glands and in peripheral blood of pSS patients (9–13). Furthermore, Tfh cell numbers are correlated to autoantibody production and disease severity, suggesting a significant role for these cells in the immunopathology of pSS (9–13).

Recent work from our group and others has implicated a role for “Tfh-like” cells in pSS immunopathology, which do not express CXCR5, but are characterized by C-C chemokine receptor 9 (CCR9) expression. CCR9+ CD4 T cells induce CD8 T cell-dependent immunopathology in mucosa-associated tissue, including salivary glands in Sjögren-like disease in mice (14). In pSS patients CCR9+ CD4 T cell numbers are increased in the salivary glands and blood compared to controls and express more IL-21, IL-4 and IFN-γ than CXCR5+ CD4 T cells (14, 15). Also, the ligand for CCR9, C-C chemokine ligand 25 (CCL25), is elevated in salivary gland tissue of pSS patients compared to controls (15). In healthy human salivary gland tissue *CCL25* mRNA is not detectable (16).

In our previous work studying CCR9+ CD4 T cells in pSS we identified genes that are specifically upregulated in CCR9+ CD4 T cells and were involved in effector T cell pathways compared to CXCR5+ and CCR9-CXCR5- CD4 T cells (17). One of the identified genes is *CCL5*. CCL5 [regulated upon activation normal T cell expressed and secreted (RANTES)] is a chemokine which mediates trafficking and homing of e.g. T cells, monocytes and NK cells. It is associated with Th1 activity, just like its receptor CCR5 (17–19). The main producers of type I IFNs, pDCs, have an elevated expression of CCR5 in pSS patients compared to healthy controls (HC) (20). Both CCL5 and CCR5 are increased in salivary glands of pSS patients (21, 22). This increase in both is also seen in inflamed glands in Sjögren-like disease in a murine model, where blocking of CCL5 reduces disease (23).

Interestingly, in previous studies CD8 T cells were found to be rapid and more potent producers of CCL5, originating from unique vesicles and released upon activation (24, 25). CD8 T cells through production of CCL5 have been shown to regulate inflammation in experimental models of arthritis. In particular CD103+ CD69+ CD8 T resident memory (Trm) cells were found in elevated numbers in previously inflamed joints in rheumatoid arthritis (RA), and shown to initiate flares by recruiting circulating lymphocytes by CCL5 secretion. Inhibition of CCL5 release by these cells inhibited arthritis flares in a murine model of RA (26).

In recent years numerous studies have shown that CD8 T cells are not merely cytotoxic killers, but can orchestrate inflammation. In this respect CXCR5+ CD8 T cells were found to display Tfh features (27). In line with these observations in a Sjögren-like disease mouse model knockdown of CD8α showed diffuse infiltration instead of focal infiltration with no acinar atrophy, duct damage, fibrosis nor leukocyte infiltration, and similar results were found after therapeutic depletion of CD8 from salivary glands, resulting e.g. in improvement of saliva production (4). Furthermore, whereas CD4 T cells predominate in lymphocytic infiltrates in the salivary glands, one subset of CD8 T cells, the CD103+CD69+ CD8 T resident memory (Trm) cells outnumber CD103+CD69+ CD4 Trm cells and show elevated IFN-γ production in Sjögren-like disease in mice (4). In pSS, circulating activated CD8 T cells correlate with a multi-omics-based pSS disease signature and HLA-DR-expressing CD8 T cells correlate to disease activity, as captured by the EULAR Sjögren’s syndrome disease activity index (ESSDAI) (28, 29). Altogether these data suggest an important role of CD8 in orchestrating pSS immunopathology.

Previously, we and others have demonstrated increased CCL25 in mucosa-associated tissues, including salivary glands of pSS patients compared to non-Sjögren sicca (nSS) patients in conjunction with increased CCR9-expression (on CD4-positive and CD4-negative T cells) (14, 15). Using CyTOF it was established that CD8 T cells are significantly increased in pSS patients with a lymphocytic focus score of 1 (LFS≥1) as compared to nSS patients without foci (LFS=0) (29). To the best of our knowledge previously no associations of CD8 T cells or CCR9+ CD8 T cells with B cell hyperactivity associated markers, including Tfh cells, in pSS and nSS patients have been reported. Given the pivotal role of CD8 T and CCR9+ T cells in Sjögren-like disease, the important pro-inflammatory role of CCL5 and CD8 T cells secreting CCL5, and the fact that CD8 T cells can display features of Tfh cells, in this study we investigated the role of CD8 T cells, and in particular CCR9+ CD8 T cells, in CCL5 secretion in pSS. To this end we studied the association of CD8 T cells with Tfh-associated lymphocytic foci. In addition, we studied the frequency of CCR9+ CD8 T cells in pSS patients versus HC, CCL5 expression and secretion of CD8 T cell subsets in pSS versus HC and CCL5 release by CD8 T cells as compared to CD4 T cells.

## Methods

### Patients and Healthy Controls

As previously described (30), for assessment of inflammatory cells, including CD8 T cells by epigenetic cell counting, salivary gland tissue was collected from n=13 non-Sjögren sicca (nSS) patients and n=29 pSS patients. For flow cytometry n=7 pSS patients and n=13 age and sex matched healthy controls (HC) were included. All patients were diagnosed by a rheumatologist. The pSS patients all met the 2016 ACR-EULAR criteria (31). The nSS patients were defined as patients with sicca complaints, who do not fulfill the classification criteria, do not have a connective tissue disease and are anti-SSA negative, with LFS=0. All patients and healthy controls were included in the University Medical Center Utrecht (UMC Utrecht). The UMC Utrecht Medical Research Ethics Committee (METC) approved the study (reference 14/589 for usage of surplus salivary gland tissue and reference 13/697 for work with peripheral blood). All participants gave written informed consent. Demographic and clinical data are presented in **Table 1**.

**Table 1:** Participants’ characteristics.

	Epigenetic cell counting		Flow cytometry	
	nSS (n=13)	pSS (n=29)	HC (n=13)	pSS (n=7)
**Female, n (%)**	10 (77)	26 (90)	13 (100)	7 (100)
**Age, years**	47 ± 14	53 ± 13	52 ± 11	55 ± 9
**Anti-Ro/SSA positive, n (%)**	0	20 (69)	–	5 (71)
**Anti-La/SSB positive, n (%)**	0	9 (31)	–	3 (43)
**ANA positive, n (%)**	4 (31)	20 (69)	–	5 (71)
**Lymphocytic focus score (foci/4mm^2^)**	0	2,3 ± 1,4	–	2,7 ± 1,5
**IgA positive plasma cells (%)**	75 ± 14	49 ± 16	–	51 ± 20
**Schirmer (mm/5min)**	4,8 ± 7,1	6,2 ± 6,7	–	0,3 ± 0,4
**Serum IgG (g/L)**	11,0 ± 2,9	16,3 ± 8,1	–	12,8 ± 2,8
**ESSDAI score (0-123)**	–	3,9 ± 3,2	–	4,1 ± 3,0
**ESSPRI score (0-10)**	–	4,7 ± 2,2	–	6,3 ± 1,1
**Immunosuppressants, n** - **Hydroxychloroquine**	3 3	8 6	–	2 1

Mean ± SD are shown unless stated otherwise. nSS, non-Sjögren sicca; pSS, primary Sjögren’s syndrome; HC, healthy controls; ESSDAI, EULAR Sjögren’s Syndrome Disease Activity Index; ESSPRI, EULAR Sjögren’s Syndrome Patient Reported Index.

### Epigenetic Cell Counting

Epigenetic cell counting was performed by Epiontis GmbH (Berlin, Germany), full methodology was as previously reported (30, 32). In brief, epigenetic cell counting quantifies cell frequencies based on cell-specific methylome markers of cellular DNA. Using bisulfite converted DNA as substrate, qPCR-assays were performed for the selected cell type-specific demethylated loci and for a locus known to be demethylated in all cell types (GAPDH). For the present analyses, data of epigenetic-based cell counts are presented as the percentage of cell-specific demethylation divided by GAPDH locus demethylation within salivary gland tissue DNA samples and multiplying that ratio by 100 (32). Thus, CD3 T cells, B cells, CD4 and CD8 T cells, as well as regulatory T cells, Tfh and Th17 cells were assessed.

### Flow Cytometry

Fresh peripheral blood mononuclear cells (PBMCs) from Lithium-heparinized blood were isolated using Ficoll density gradient centrifugation. Fresh PBMCs were first stained with fixable viability dye eF780 (eBioscience) for 10 minutes at 4°C and after washing the cells were incubated with fluorochrome-conjugated antibodies against CD3, CD4, CD8, CCR9, CCL5, CD27, CD45RO, IFN-γ and TNF-α for 25 minutes at 4°C (details of used antibodies are listed in **Supplementary Table 1**. Gating strategies are shown in **Supplementary Figure 1**). Intracellular staining of CCL5, IFN-γ and TNF-α was performed according to the Fixation-Permeabilization protocol from the manufacturer (eBioscience) after 4 hours incubation with phorbol myristate acetate (PMA), ionomycin and Brefeldin A. All samples were measured on a BD LSRFortessa using DIVA software version 8.0.1. Analysis of the obtained FCS files was performed using FlowJoTM Software (BD Life Sciences). It was previously shown that CCL5 release is not blocked by Golgi complex blockade, such as Brefeldin A and subsequently reduced expression is a consequence of increased release (24, 25). A medium control was taken along to calculate CCL5 release. The release was defined by the difference between CCL5 expression in the medium condition minus CCL5 expression after stimulation (deltaCCL5).

### Statistical Analysis

Mann-Whitney U test (for comparison between pSS and HC) and Wilcoxon-non parametrical paired test were used. For correlations Pearson’s correlation and Spearman’s rho were used where appropriate. Data were analyzed using FlowJoTM Software, Graphpad Prism 8, IBM SPSS Statistics 26. Statistical significance was considered for differences at p ≤ 0.05.

## Results

### CD8 T Cells Are Abundantly Present in Salivary Glands in pSS and Correlate With Lymphocytic Foci and Tfh Cell Numbers

**Figure 1:**
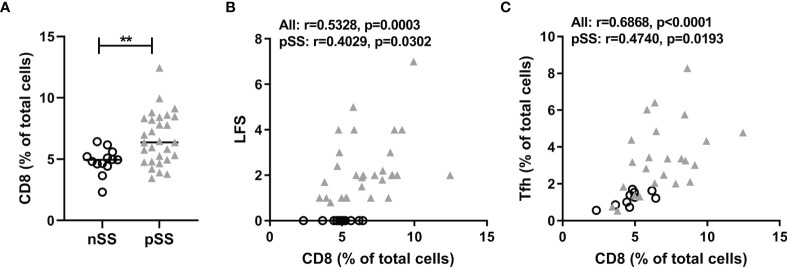
CD8+ T cells are abundantly present in salivary glands in pSS and correlate with lymphocytic foci and T follicular helper cell numbers. Epigenetic cell counting from labial salivary gland tissue DNA was used to determine **(A)** the percentage of CD8 T cells in nSS versus pSS patients. **(B)** Spearman’s correlation between CD8 T cell percentages and lymphocytic focus scores (LFS). **(C)** Spearman’s correlation between CD8 T cell percentages and Tfh cell percentages. nSS, non-Sjögren sicca; pSS, primary Sjögren’s syndrome; LFS, lymphocytic focus score; Tfh, T follicular helper. ** indicates statistical significance of 0.01.

We performed new analyses on epigenetic cell counting data of tissue DNA from labial salivary gland tissue from nSS and pSS patients. So far we have not been able to assess CCR9-specific CD8 T cell counts in tissue due to technical limitations. However, we were able to assess percentages of CD8 T cells, which were significantly higher in pSS patients compared to nSS patients (**Figure 1A**). Furthermore the percentage of CD8 T cells in the salivary gland tissue significantly correlated with the lymphocytic focus score (LFS), a hallmark clinical parameter in pSS (**Figure 1B**). Also, CD8 T cell numbers significantly correlated with Tfh cell numbers (**Figure 1C**). Interestingly, significant correlations were also found between CD8 T cell and both B cell numbers (Spearman’s rho=0.44 p<0.01) and serum IgG (Spearman’s rho=0.45, p<0.01) (**Supplementary Figure 2**). Finally, increased CD8 T cell numbers correlated with reduced percentages of IgA found in the salivary gland tissue which is a consequence of increased IgG and IgM-producing plasma cells (Spearman’s rho=-0.41, p<0.01, **Supplementary Figure 2**).

### CD8 Effector T Cells, in Particular CCR9+ CD8 Effector T Cells, Express High Levels of CCL5 That Is Reduced in pSS Patients as Compared to Healthy Controls

**Figure 2:**
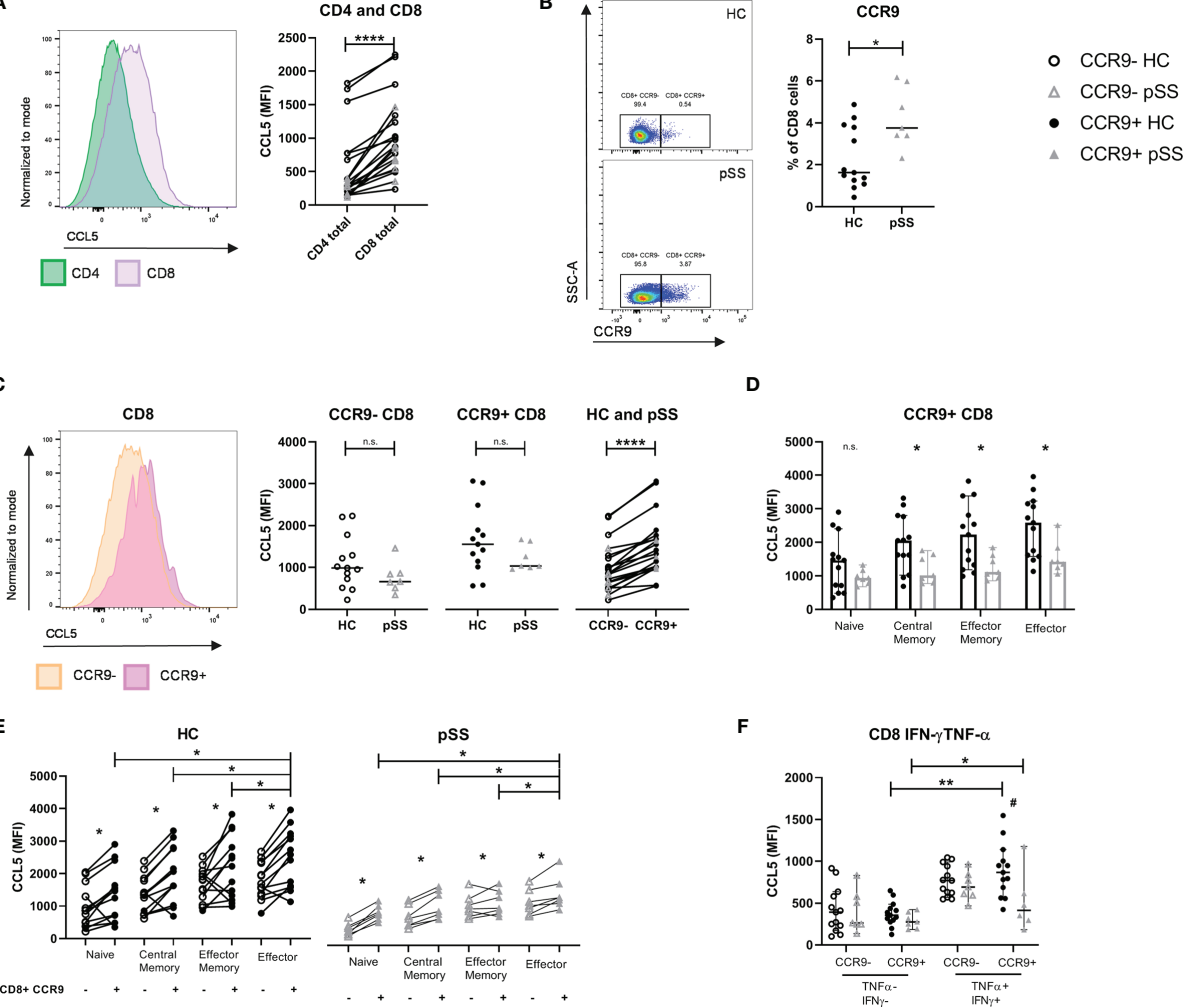
CCL5 expression is highest in CCR9+ CD8 T cells, of which pSS patients have significantly higher cell numbers. Memory and effector CCR9+ cells from pSS patients express reduced CCL5 levels compared to HC. Flow cytometric analyses comparing for HC (n=13) and pSS patients (n=7). **(A)** CCL5 expression on CD4 versus CD8 T cells. **(B)** Percentage of CCR9-expressing CD8 T cells from PBMCs of HC and pSS (representative plots of CCR9 expression from a HC and a pSS patient). **(C)** CCL5 expression compared for CCR9- and CCR9+ CD8 T cells in HC and pSS patients and between CCR9- and CCR9+ CD8 T cells. **(D)** CCL5 expression in CD27/CD45RO-defined cell subsets within the CCR9+ CD8 T cells. MFI depicted as median with 95% confidence interval. CCL5 expression for HC and pSS comparing CCR9- and CCR9+ CD27/CD45RO-defined CD8 T cell subsets **(E)** and in IFN-γ/TNF-α double negative and double positive CCR9- and CCR9+ T cells **(F)**. Representative histograms show CCL5 expression normalized to mode. Naive: CD27+CD45RO-; Central Memory: CD27+CD45RO+; Effector Memory: CD27-CD45RO+; Effector: CD27-CD45RO-. MFI, mean fluorescence intensity; pSS, primary Sjögren’s syndrome; HC, healthy controls; PBMCs, peripheral blood mononuclear cells; n.s., not significant. *, **, **** indicates statistical significance of p<0.05, 0.01, 0.0001, respectively. # indicates significant difference with p<0.05 between pSS and HC in **(F)**.

After confirming the local abundance of CD8 T cells in salivary gland tissue of pSS patients we next determined the expression of CCL5 in circulating CD8 and CD4 T cells. We found higher expression of CCL5 in CD8 T cells compared to CD4 T cells (**Figure 2A**). In addition, increased percentages of CCR9+ CD8 T cells were observed in pSS patients compared to HC (**Figure 2B**). pSS patients with anti-SSA-antibodies in particular had higher percentages of CCR9+ CD8 T cells compared to HC [median 4.7 (IQR 3.4 – 5.9) for pSS patients and median 1.6 (IQR 1.3 – 3.8) for HC, p=0.03], which was not observed for SSA- patients [median 3.6 (IQR 3.5-3.7)]. In this relative small cohort no significant correlations of CCR9+ CD8 T cells with ESSDAI, sIgG, and LFS were found.

CCR9+ CD8 T cells expressed higher levels of CCL5 compared to CCR9- CD8 T cells. When analyzing total CD8 T cells no significant difference was seen in CCL5 expression in pSS versus HC (**Figure 2C**). However, when analyzing CD27/CD45RO-defined cell compartments memory and effector CD8 T cells from pSS patients were shown to have significantly reduced CCL5 expression as compared to HC (**Figure 2D** shows the results for CCR9+ CD8 T cells, and **Supplementary Figure 3** the results for CCR9- CD8 T cells and CD8 total).

Both in HC and pSS patients the CCL5 expression is increased in antigen-experienced cells. CCR9+ effector CD8 T cells express the highest level of CCL5 (compared to naive, central memory and effector memory cells, **Figure 2E**). Similar results are found when defining effector profile based on IFN-γ/TNF-α co-expression. A significantly higher expression of CCL5 is found in IFN-γ+TNF-α+(double positive) CCR9+ CD8 T cells as compared to IFN-γ-TNF-α-(double negative) CCR9+ CD8 T cells. Just as for CD27/CD45RO-defined effector cells, IFN-γ+TNF-α+ (double positive) CCR9+ CD8 T cells from pSS patients express significantly less CCL5 compared to HC (**Figure 2F**).

### Effector (Memory) Cells Have the Highest Capacity to Release CCL5 in HC and This Release Is Reduced in pSS Patients

**Figure 3:**
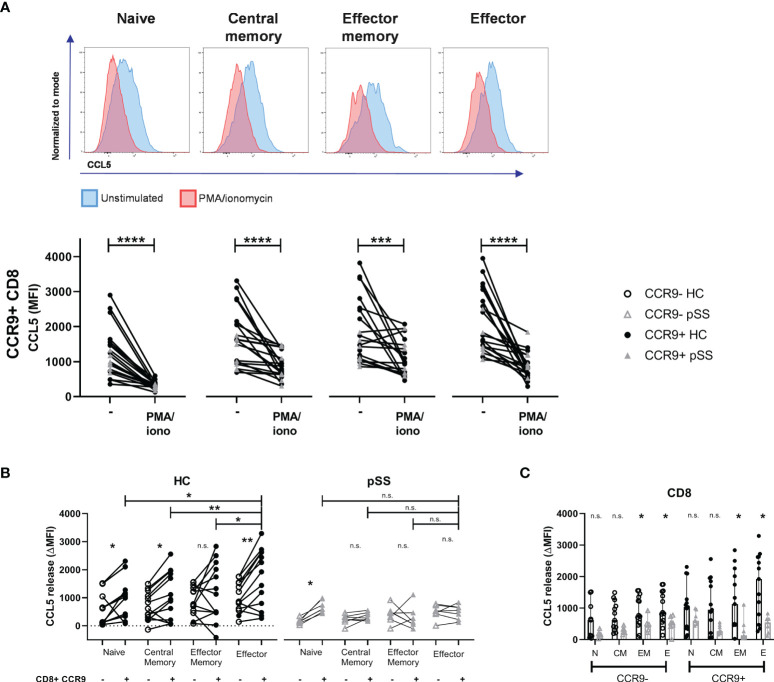
Effector (memory) cells of pSS patients release less CCL5 after stimulation. CCR9+ CD8 T cells from HC release more CCL5 than CCR9- CD8 T cells, which is highest in effector cells. CD27/CD45RO-defined CD8 T cell subsets from n=13 HC and n=7 pSS were analyzed using flow cytometry. **(A)** Representative histograms show CCL5 expression (normalized to mode) of unstimulated (blue) versus PMA/iono stimulated (red) cells with below quantification for CCR9+ CD8 T cells. **(B)** Difference in CCL5 expression (ΔMFI CCL5) between unstimulated and stimulated CD8 T cells. Graph shows differences in CCR9- and CCR9+ CD8 T cell subsets, both for HC and for pSS patients. **(C)** Bar plot showing differences between HC and pSS in ΔMFI CCL5 in CCR9- and CCR9+ CD8 T cell subsets. Naive (N): CD27+CD45RO-; Central Memory (CM): CD27+CD45RO+; Effector Memory (EM): CD27-CD45RO+; Effector (E): CD27-CD45RO-. MFI, mean fluorescence intensity; ΔMFI, deltaMFI (CCL5 difference of 4h unstimulated-4h stimulated); pSS, primary Sjögren’s syndrome; HC, healthy controls; PMA/iono, phorbol myristate acetate/ionomycin. *, **, ***, **** indicates statistical significance of p<0.05, 0.01, 0.001, 0.0001, respectively. n.s., not significant.

In order to evaluate CCL5 release we compared the medium condition to 4 hours stimulation with PMA/ionomycin. Upon this stimulation CD8 T cells express less CCL5 compared to unstimulated cells (**Figure 3A**). In HC a significant difference was seen in CCL5 release between CCR9- and CCR9+ CD8 T cells in all subsets except for the effector memory subset. CCR9+ effector CD8 T cells released most CCL5 compared to the other subsets. Associated with lower CCL5 expression levels in pSS memory and effector CD8 T cell subsets (as compared to HC - **Figure 2D**), these differences in CCL5 release were not seen for the pSS patients. In contrast, naive CD8 T cells that did not significantly differ in CCL5 expression from HC *ex vivo*, showed significantly different CCL5 release upon stimulation in both HC and pSS patients (**Figure 3B**).

No significant differences in CCL5 release from naive and central memory CD8 T cell subsets were found between pSS patients and HC. From effector memory and effector CD8 T cells from pSS patients less CCL5 release was detected than from HC (**Figure 3C**).

### CCR9- and CCR9+ CD8 T Cells Release CCL5 Equally Effective and Rapidly, but CCR9+ CD8 T Cells Release Significantly More CCL5 Within 10 Minutes Than CCR9+ CD4 T Cells

**Figure 4:**
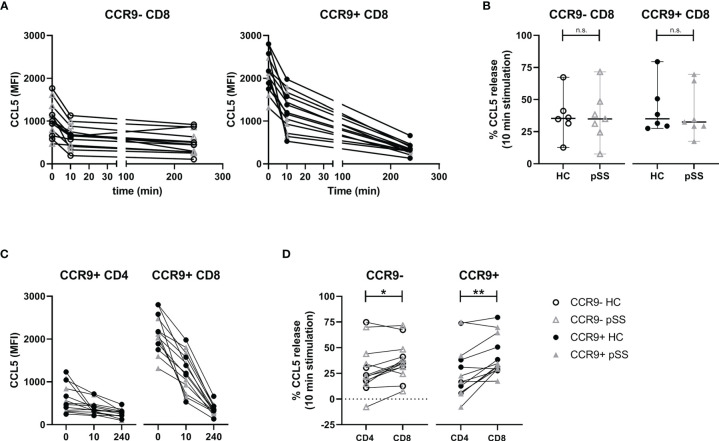
Rapid relative but not absolute CCL5 release is comparable between HC and pSS. CCR9+ CD8 T cells release more CCL5 than CCR9+ CD4 T cells. For n=6 HC and n=7 pSS the MFI CCL5 was determined at t=0, t=10 minutes and t=4 hours. **(A)** Graphs showing MFI CCL5 over time for both CCR9- and CCR9+ CD8 T cells. **(B)** Comparison of CCL5 release after 10 minutes stimulation with phorbol myristate acetate (PMA) and ionomycin between HC and pSS patients in CCR9- and CCR9+ CD8 T cells. **(C)** Graphs depicting timepoints t=0, t=10m and t=4h (unscaled) for CCR9+ CD4 T cells and CCR9+ CD8 T cells combining HC and pSS patients. **(D)** difference between CD4 and CD8 T cells in CCL5 release after 10 minutes stimulation for CCR9- and CCR9+ cells. MFI, mean fluorescence intensity; pSS, primary Sjögren’s syndrome; HC, healthy controls; n.s., not significant. *, **, indicates statistical significance of p<0.05, 0.01, respectively.

To assess the pace of release we compared *ex vivo* CCL5 expression with 10 minutes and 4 hours of PMA/ionomycin stimulation. This showed neither relative differences between CCR9- and CCR9+ CD8 T cells, nor differences between pSS patients and HC (**Figure 4A, B**), but when looking at mean fluorescence intensities (MFI) CCR9+ CD8 T cells displayed the highest release (median MFI decrease 419 and 995 for CCR9+ CD8 T cells versus 258 and 278 for CCR9- CD8 T cells at 10 minutes and 4 hours respectively).

When comparing with CCR9+ CD4 T cells also no differences in CCL5 release were found between pSS and HC (**Figure 4C**). Yet, there was a difference in release within 10 minutes, with CCR9+ CD8 T cells releasing significantly more CCL5 than CCR9+ CD4 T cells (**Figure 4D**, median 33% [IQR 29-51] and 17% [IQR 7-38], respectively). This corresponds to a decrease in median MFI of 419 in CCR9+ CD8 T cells versus a decrease of 68 in CCR9+ CD4 T cells.

## Discussion

This study demonstrates that CD8 T cells are elevated in the salivary gland tissue of pSS patients compared to nSS patients and that their numbers are correlated to the lymphocytic aggregates and the number of Tfh cells and B cells. Activation induces release of CCL5, which is reflected by reduced intracellular CCL5 levels. CD8 T cells secrete higher CCL5 levels than CD4 T cells, with the highest levels found in and secreted by effector CD8 T cells, in particular those cells expressing CCR9. The frequency of CCR9+ CD8 T cells is increased in pSS patients. CCL5 expression and subsequently release of CCL5 upon *in vitro* activation was higher in circulating CD8 T cells from healthy controls than pSS patients.

Using epigenetic cell counting we found elevated numbers of CD8 T cells in salivary gland tissue of pSS patients compared to nSS patients. The CD8 T cell presence in lymphocytic infiltrates of pSS patients has been implicated in salivary gland dysfunction, possibly by inducing apoptosis of acinar epithelial cells (33). Many of the CD8 T cells in the salivary glands of pSS patients are considered Trm cells, expressing both CD103 and CD69 (4, 33). Whereas CD8 Trm cells could serve a “traditional” CD8 direct cytotoxic role, it has also been suggested that they rather regulate local inflammation by producing cytokines, but are not terminally differentiated and can e.g. gain properties fitting to memory T cells from the circulation (34, 35). Another indication towards a more orchestrating CD8 function is found in “Tfh-like” CD8 T cells as have been described in Hodgkin lymphoma. CXCR5+ICOS+ CD8 T cells were found secreting cytokines such as IL-4, IL-21 and CXCL13, whereas little to no IFN-γ, perforin or Granzyme B was secreted (27). In our data we find significant correlations between CD8 T cell numbers and Tfh and B cell numbers in the salivary glands, the lymphocytic focus score and percentage of IgA+ cells in the lymphocytic aggregate and serum IgG levels. Although we cannot discriminate whether CD8 T cells, e.g. by secretion of CXCL13, contribute to formation of the foci, the strong cytokine producing potential in this study strongly suggests that they could play a key role.

Earlier work in our group has shown that CCR9+CXCR5- “Tfh-like” CD4 T cells form a distinct group of cells, which show a potent pro-inflammatory phenotype and have an increased frequency in peripheral blood and salivary gland tissue of pSS patients (15). Chemokine CCL5 was identified as a potential target, and was highest expressed by effector CCR9+ CD4 T cells (17). CD8 T cells, however, are described as a more potent producer of CCL5 (36, 37): a finding we confirmed in this study for both HC and pSS patients. We found that CCR9+ CD8 T cells and particularly those with an effector (memory) profile have the highest CCL5 levels. As prolonged T cell stimulation reduces CCR9 expression, we have experienced difficulties in finding CCR9 expression and mapping CCL5, because of its rapid release is challenging. Hence, the expression and release of CCL5 by (CCR9-expressing) CD8 T cells in the inflamed glands remains to be established. However, in juvenile idiopathic arthritis (JIA) isolated synovial CD8 T cells were found to rapidly release high levels of CCL5 upon TCR stimulation, without new protein synthesis, and synovial CD8 T cells showed high levels of CCL5 protein and increased CCL5 mRNA as compared to CD8 T cells from peripheral blood (38). In analogy to rheumatoid arthritis (RA) synovial fluid T cells, which were shown to be almost completely antigen-experienced memory/effector cells (39), this suggests that in the salivary glands of pSS patients the results we have found in blood will be even more pronounced. In support of this we demonstrated that effector CD8 T cells were rapid and high CCL5 producers.

Although CCR9+ effector CD8 T cells expressed the highest CCL5 levels, also in all other CD27/CD45RO-defined CD8 T cell subsets a significantly higher CCL5 expression was found in CCR9+ cells as compared to CCR9- CD8 T cells, which we previously also demonstrated for CD4 T cells (17). Since CCR9 is a chemokine receptor that initiates migration of T cells towards CCL25 under both physiological and inflammatory conditions and CCL5 is a chemokine that recruits several types of leukocytes to inflamed sites, the high expression of CCL5 in CCR9-expressing cells indicates that facilitating recruitment of other inflammatory cells is a main feature of these cells in immunopathology.

Between pSS patients and controls several significant differences in CCL5 expression and release were found. Memory and effector CCR9+ CD8 T cells from pSS patients have a significantly lower CCL5 expression than from HC. Also, the subsequent release of CCL5 by these cells is lower in pSS patients than in controls. However, since after 10 minutes of stimulation the relative release of CCL5 is similar between pSS patients and controls, this indicates that CCR9-expressing cells in pSS patients did not lose the ability to quickly release CCL5. Together, these results could indicate that in the proinflammatory environment of pSS some CCL5 is already released by CD8 T cells, yet these cells retain their ability to release CCL5 and are not exhausted. Together these findings match the increased levels of CCL5 in pSS patients. One study has found elevated serum CCL5 in pSS patients with an elevated ESR (40). In addition, several studies have shown increased expression of CCR5 by pDCs, cDC2s, and CCR9+ CD4 T cells (17, 20). Both CCL5 and CCR5 were shown to be increased in salivary glands of pSS patients and thus increased release of CCL5 by CD8 T cells could play a key role in regulation of chemotaxis and subsequent inflammation (21, 22). This is supported by the fact that blockade of CCL5 in inflamed glands halts Sjögren-like disease and that blockade of CCL5 by CD8 T cells halts experimental arthritis (23, 26).

What exactly triggers the release of CCL5 is unknown, but several autoantigens have been suggested to initiate T cell activation in pSS and could trigger CCL5 release (41). In addition, several viruses such as EBV and HTLV-1 have been demonstrated in pSS and could trigger inflammation (42–44). Additionally, induction of cytokines such as IL-7 and IL-15, which are known to be upregulated in the glands of pSS patients, could induce cytokine-activated T cells and trigger release of CCL5. Such cytokines could arise upon innate triggering or following stimulation by type I and type II IFNs (45, 46). Our data show that increased expression of CCL5 could arise from increased frequencies of effector T cells co-expressing IFN-γ and TNF-α, and specifically those co-expressing CCR9. The rapid release of CCL5 by CD8 T cells in all cases could play an important role in the initiation of the immunopathology in pSS.

Besides differences in CCL5 expression also the difference in CCR9+ CD8 T cell numbers stands out. Just as for CCR9+ CD4 T cells, increased percentages of CCR9+ CD8 T cells were observed in pSS patients compared to healthy controls. To the best of our knowledge this is the first paper demonstrating that CCR9-expressing CD8 T cell numbers are elevated in pSS patients. Considering the increased expression of CCL25 in the inflamed salivary glands this suggests that similarly to CCR9+ CD4 T cells the increased CCR9+ CD8 T cells could home to the glands. In addition, we found a very strong correlation between Tfh cells and CXCL13 (30). Interestingly, we recently also demonstrated co-expression of CCR9 and CXCR5 on a small percentage of circulating CD8 T cells (manuscript in preparation). This suggests that at least a proportion of CCR9+ CD8 T cells can migrate to the lymphocytic infiltrates. These cells through release of CCL5 could further stimulate inflammation.

Even though our study is performed on samples from a small number of participants, our results clearly indicate differences between pSS patients and controls, not only in frequency of CCR9-expressing CD8 T cells, but also in these cells’ expression and release of CCL5. Given the well-described strong recruiting abilities of CCL5 and the potent anti-inflammatory effect of targeting CCL5, in particular CD8-produced in several experimental models, this might present a new factor that plays a key role in pSS immunopathology.

## Data Availability Statement

The original contributions presented in the study are included in the article/**Supplementary Material**. Further inquiries can be directed to the corresponding author.

## Ethics Statement

The studies involving human participants were reviewed and approved by the Medical Research Ethics Committee of the University Medical Center Utrecht (METC no.13/697). The patients/participants provided their written informed consent to participate in this study.

## Author Contributions

AH, SB, AK, FL, HL, and JR were involved in conception and design of the study. AH, SB, AK, and HL were involved in data acquisition. AH, SB, AK, FL, HL, and JR were involved in data analysis and interpretation. AH drafted the manuscript. All authors revised the manuscript critically and approved the final version of the manuscript to be published.

## Funding

AH is supported by ReumaNederland: grant number 17-2-403.

## Conflict of Interest

The authors declare that the research was conducted in the absence of any commercial or financial relationships that could be construed as a potential conflict of interest.

## Publisher’s Note

All claims expressed in this article are solely those of the authors and do not necessarily represent those of their affiliated organizations, or those of the publisher, the editors and the reviewers. Any product that may be evaluated in this article, or claim that may be made by its manufacturer, is not guaranteed or endorsed by the publisher.
