# Supplementary material for: CCL5 Release by CCR9+ CD8 T Cells: A Potential Contributor to Immunopathology of Primary Sjögren’s Syndrome
Source: Front Immunol. 2022 Jun 1;13:887972. doi: 10.3389/fimmu.2022.887972 (PMC9198220; doi:10.3389/fimmu.2022.887972)
Supplement: Supplementary file 1 [file DataSheet_1.docx]

**Supplementary Table 1. Antibodies used for flow cytometry.**

| Marker | Fluorochrome | Company | Clone |
| --- | --- | --- | --- |
| CD3 | AF700 | Sony Biotechnology | UCHT1 |
| CD4 | BV785 | Biolegend | RPA-T4 |
| CD8 | FITC | BD Biosciences | RPA-T8 |
| CCR9 | APC | Biolegend | L053E8 |
| CCL5 | PE | Biolegend | VL1 |
| CD27 | BV510 | BD Biosciences | L128 |
| CD45RO | PE-Cy7 | BD Biosciences | UCHL1 |
| IFN-gamma | PerCP-Cy5.5 | eBioscience | 4S.B3 |
| TNF-alpha | BV421 | BD Biosciences | Mab11 |
| Fixable Viability Dye | eF780 | eBiosciences | n.a. |


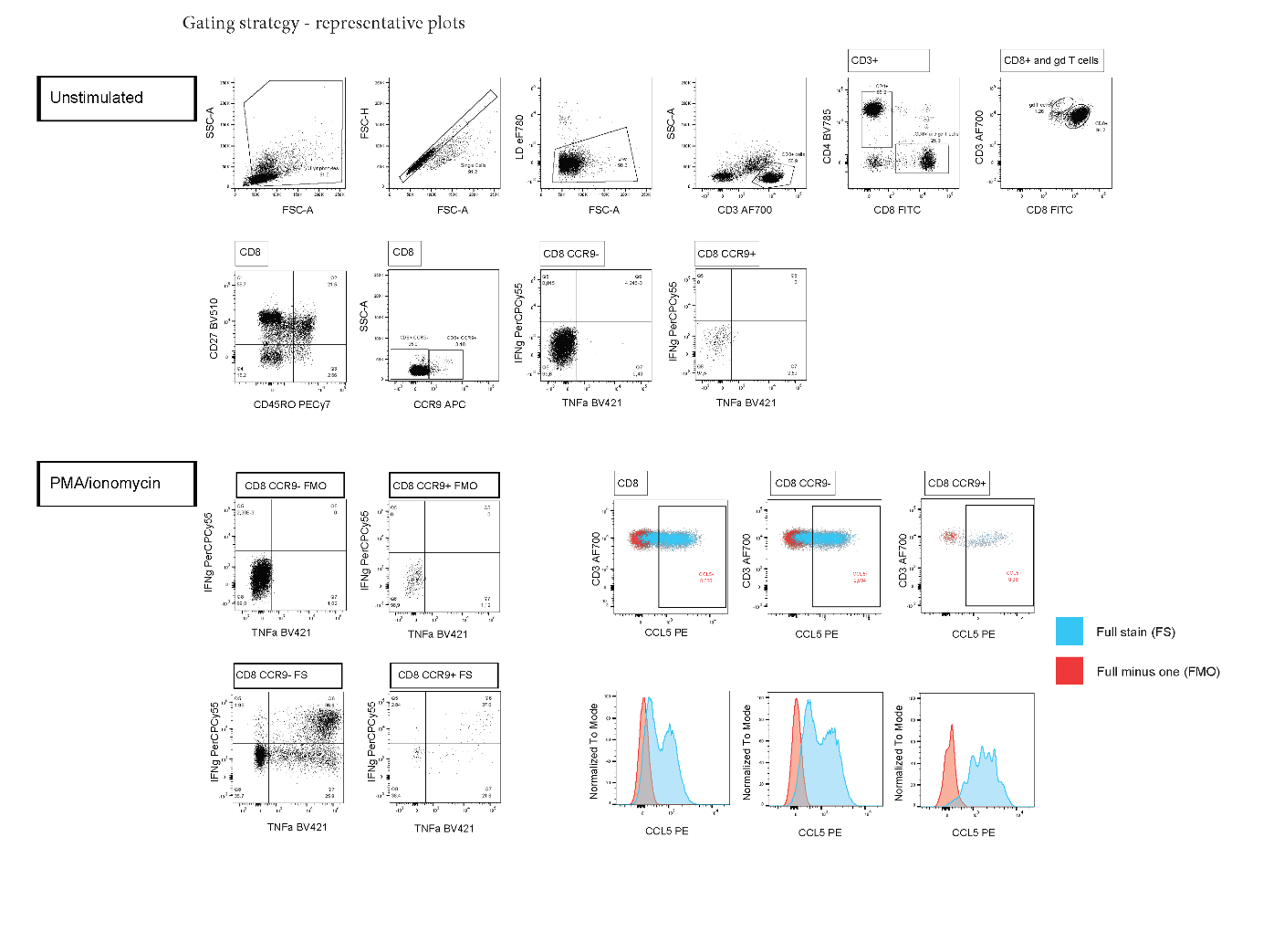
**Supplementary Figure 1. Gating strategy.** Upper two rows show representative flow cytometry (intracellular) staining gating strategies of unstimulated cells; bottom two rows of PMA/ionomycin-stimulated cells. Both conditions shown are after 4 hours of culture.

Top (first) row, panels left to right: forward versus side scatter (FSC vs SSC); selection of single cells, selection of live cells using fixable viability dye; selection of CD3+ cells, followed by selection of CD8+ single positive cells. Top second row: CD27/CD45RO staining of CD8+ cells, CCR9-expression on CD8+ T cells and intracellular IFN-γ/TNF-α expression in unstimulated on CCR9- and CCR9+ CD8+ T cells.

Bottom first row, from left to right: PMA/ionomycin-stimulated cells with FMO for both IFN-γ and TNF-α in CCR9- and CCR9+ CD8 T cells, with below the respective full stained samples.

Bottom, colored plots, from left to right: dotplot overlay and below histograms of CCL5 expression in given subsets comparing full stain with FMO stain.

**Supplementary Figure 2. CD8 T cell number correlates to percentage IgA and B cell number in salivary gland tissue, and to serum IgG.** Epigenetic cell counting from labial salivary gland tissue DNA was used to determine (A) Spearman’s correlation between CD8 T cell percentages and percentage of IgA positive cells in salivary gland tissue. (B) Spearman’s correlation between CD8 T cell percentages and B cell percentages. (C) Spearman’s correlation between CD8 T cell percentages and level of IgG in serum (in g/L). nSS: non-Sjögren sicca; pSS: primary Sjögren’s syndrome; SG: salivary gland; sIgG: serum IgG.

**Supplementary Figure 3. MFI CCL5 pSS vs HC in subsets.**

Flow cytometric analyses comparing for HC (n=13) and pSS patients (n=7). CCL5 expression in pSS patients and HC in CD27/CD45RO-defined cell subsets (A) within both CCR9- and CCR9+ CD8 T cells and (B) within CD8 total. MFI depicted as median with 95% confidence interval. Naive: CD27+CD45RO-; Central Memory: CD27+CD45RO+; Effector Memory: CD27-CD45RO+; Effector: CD27-CD45RO-.MFI: mean fluorescence intensity. pSS: primary Sjögren’s syndrome; HC: healthy controls. n.s.: not significant. *, **, ***, **** indicates statistical significance of p<0.05, 0.01, 0.001, 0.0001, respectively.
